# Employing external facilitation to implement cognitive behavioral therapy in VA clinics: a pilot study

**DOI:** 10.1186/1748-5908-5-75

**Published:** 2010-10-13

**Authors:** Michael R Kauth, Greer Sullivan, Dean Blevins, Jeffrey A Cully, Reid D Landes, Qayyim Said, Thomas A Teasdale

**Affiliations:** 1 South Central Mental Illness Research, Education and Clinical Center (MIRECC), Department of Veterans Affairs, Fort Roots Drive, Little Rock, AR, USA; 2 Michael E. DeBakey VA Medical Center, Holcombe Boulevard, Houston, TX, USA; 3 Menninger Department of Psychiatry & Behavioral Sciences, Baylor College of Medicine, One Baylor Plaza, Houston, TX, USA; 4 Central Arkansas Veterans Healthcare System, Fort Roots Drive, Little Rock, AR, USA; 5 University of Arkansas for Medical Sciences, Markham Street, Little Rock, AR, USA; 6 Houston Center for Quality of Care and Utilization Studies, Holcombe Boulevard, Houston, TX, USA; 7 Veterans Affairs Medical Center, Oklahoma City, 13th Street, OK, USA; 8 University of Oklahoma Health Science Center, 13th Street, Oklahoma City, OK, USA

## Abstract

**Background:**

Although for more than a decade healthcare systems have attempted to provide evidence-based mental health treatments, the availability and use of psychotherapies remains low. A significant need exists to identify simple but effective implementation strategies to adopt complex practices within complex systems of care. Emerging evidence suggests that facilitation may be an effective integrative implementation strategy for adoption of complex practices. The current pilot examined the use of external facilitation for adoption of cognitive behavioral therapy (CBT) in 20 Department of Veteran Affairs (VA) clinics.

**Methods:**

The 20 clinics were paired on facility characteristics, and 23 clinicians from these were trained in CBT. A clinic in each pair was randomly selected to receive external facilitation. Quantitative methods were used to examine the extent of CBT implementation in 10 clinics that received external facilitation compared with 10 clinics that did not, and to better understand the relationship between individual providers' characteristics and attitudes and their CBT use. Costs of external facilitation were assessed by tracking the time spent by the facilitator and therapists in activities related to implementing CBT. Qualitative methods were used to explore contextual and other factors thought to influence implementation.

**Results:**

Examination of change scores showed that facilitated therapists averaged an increase of 19% [95% CI: (2, 36)] in self-reported CBT use from baseline, while control therapists averaged a 4% [95% CI: (-14, 21)] increase. Therapists in the facilitated condition who were not providing CBT at baseline showed the greatest increase (35%) compared to a control therapist who was not providing CBT at baseline (10%) or to therapists in either condition who were providing CBT at baseline (average 3%). Increased CBT use was unrelated to prior CBT training. Barriers to CBT implementation were therapists' lack of control over their clinic schedule and poor communication with clinical leaders.

**Conclusions:**

These findings suggest that facilitation may help clinicians make complex practice changes such as implementing an evidence-based psychotherapy. Furthermore, the substantial increase in CBT usage among the facilitation group was achieved at a modest cost.

## Background

Overall efforts to increase the provision of evidence-based mental health (MH) treatments by intervening with providers to change practices have been met with modest success [1-3], although more recent intensive efforts appear promising [4]. Clearly, training alone is insufficient to effect significant and sustainable practice change [5]. The literature identifies a number of effective provider-focused intervention strategies that have been used in healthcare dissemination and implementation efforts, including reminders, academic detailing, interactive quality-improvement workshops, local opinion leaders, and performance monitoring and feedback [6]. Interventions at the financial (*e.g.*, capitation, incentives) and organizational levels (*e.g.*, changes in technology, decision-support tools) have also been shown to be effective [7]. In general, studies have found that combinations or packages of interventions delivered simultaneously at multiple levels have been more successful in producing sustained practice change than single interventions [7]. Further, because barriers to implementation of a new practice tend to differ by site and by individuals within sites [8,9], combinations of interventions tailored to the site may be more effective in addressing different barriers at different sites and at different times. Thus, for multi-site projects, such as interventions within a complex system of care, it may be important to include both general and focused interventions for provider or site-specific problems as needed [10].

Facilitation has emerged recently as a promising integrative implementation strategy in quality-improvement and health services research. Facilitation, in this context, refers to 'the process of enabling (making easier) the implementation of evidence into practice' within a complex system of care [11]. The facilitator is an implementation expert who is either external or internal to the agency and works with individuals or teams to help them identify and solve problems around change efforts. The facilitator employs a number of strategies as needed to support the individuals or teams in their change efforts [12]. Stetler *et al. *[10] noted that two key functions of a facilitator are interactive problem solving and providing interpersonal support in the context of a quality-improvement process. The techniques employed by the facilitator, and when and in what settings, have not been clearly defined and vary across individuals and settings. Nevertheless, in four of five randomized controlled studies, facilitation has shown a modest-to-strong effect on adoption of new clinical procedures, such as health screenings, monitoring procedures, and motivational interviewing across diverse settings [13-17].

However, most studies of facilitation have focused on adding new health screenings or monitoring procedures or providing additional health counseling. Little data exist about the effect of facilitation on the adoption of complex skills and behaviors, such as a psychotherapy, that can require substantial changes in routine or established clinical processes. Descriptive studies suggest that facilitation may be beneficial for complex practice changes. Stetler *et al. *[10] described the important integrative role of external facilitation for implementation coordinators in six large Department of Veterans Affairs (VA) Quality Enhancement Research Initiative projects involving multiple interventions (*e.g.*, opinion leaders, clinical reminders, patient interventions, templated orders, feedback mechanisms, policy changes, *et al.*) at multiple sites. Although coordinators were familiar with the concept of facilitation from the PARiHS (Promoting Action on Research in Health Services) framework [18], the critical role of facilitators only emerged during the course of these projects. Stetler *et al. *[10] found that effective facilitators communicated frequently with local targets, attended occasional face-to-face meetings, actively provided encouragement and feedback, and functioned as problem solvers and mentors when necessary. In addition, Sullivan *et al. *[19] described how both external and internal facilitators aided and motivated 16 VA clinicians to implement new psychosocial rehabilitation services at eight of nine facilities after intensive training. Clinicians interviewed at the end of the study identified the facilitators as key supports in their successful application of new skills and establishment of new services.

In the past decade, many healthcare systems have engaged in efforts to increase the use of evidence-based MH treatments, including psychotherapies [4]. Although effective MH treatments are available, they are not reaching most individuals with a mental illness. In a national representative household survey in the U.S., only 41% of individuals who were diagnosed with a MH disorder received any MH services in a 12-month period [20]. Most individuals were treated for their mental illness by a general medical practitioner. Of those treated in specialty MH programs, about one-half (48.3%) received minimally adequate treatment, defined as either an appropriate medication plus more than four follow-up visits or eight or more psychotherapy sessions of 30 minutes or longer. Similarly, in a recent study using administrative databases of MH service use in the VA, only 22% of outpatients newly diagnosed with depression, anxiety, or post-traumatic stress disorder received at least one session of psychotherapy within 12 months of diagnosis, and only 4% received eight or more sessions [21]. A follow-up study found that rural veterans were even less likely than urban veterans to receive any psychotherapy, and when they did get psychotherapy, urban veterans received about twice as many sessions as rural veterans [22].

To increase use of evidence-based psychotherapies (EBPs), first, therapists must receive effective training in the therapy, gain new skills, and become clinically proficient. Second, these new practices must be implemented in routine clinical practice. Given multiple obstacles, support for implementation is necessary for sustained adoption of EBPs in routine care. There is a great need, therefore, for relatively simple but effective implementation strategies that can improve adoption in diverse healthcare settings.

The objective of this pilot study was to examine the effect of facilitation on the implementation of cognitive behavioral therapy (CBT), an evidence-based therapy, in VA clinics. We hypothesized that: therapists at sites that received facilitation would show a greater increase in CBT use from baseline to follow up, compared with therapists who received training alone; within the facilitation group more contact with the facilitator would be related to increased use of CBT; and the costs of facilitation would be relatively modest. We also examined predictors (demographics, previous training, attitudes toward evidence-based practices) of CBT use among therapists and explored the relationship between contextual differences at the facility level and adoption of CBT. To our knowledge, this is the first controlled study of external facilitation for implementation of psychotherapy.

The decision to make facilitation our primary intervention was informed by our earlier work to use facilitation to promote MH clinicians' application of skills after training [19] and by two conceptual frameworks. Our earlier study employed the Fixsen model [8] to frame the findings. In this model, key implementation 'drivers' include consultation and coaching (a form of facilitation), which we viewed as critical to our outcomes. The PARiHS framework takes the concept of facilitation even further. This framework posits in part that successful implementation is a function of the nature of facilitation to adopt the new practice and the context in which the new practice will occur, such as the extent that clinicians value the evidence for an innovation as well as the extent that organizational structures and process support practice change [18,23]. Here, facilitation is viewed as an active strategy by implementation experts to help change agents and the system make change easier. Consistent with this framework, we expected facilitation to support clinicians in quickly adopting CBT by encouraging early attempts and addressing barriers to use CBT (*e.g.*, clinic scheduling, supervisor support, *et al.*). We also expected that organizational issues unique to each site could present barriers to CBT use, and so we attempted to engage clinical leadership in the planning and implementation of the intervention in order to quickly identify and resolve systems obstacles.

## Methods

### Sites and participants

**Figure 1:**
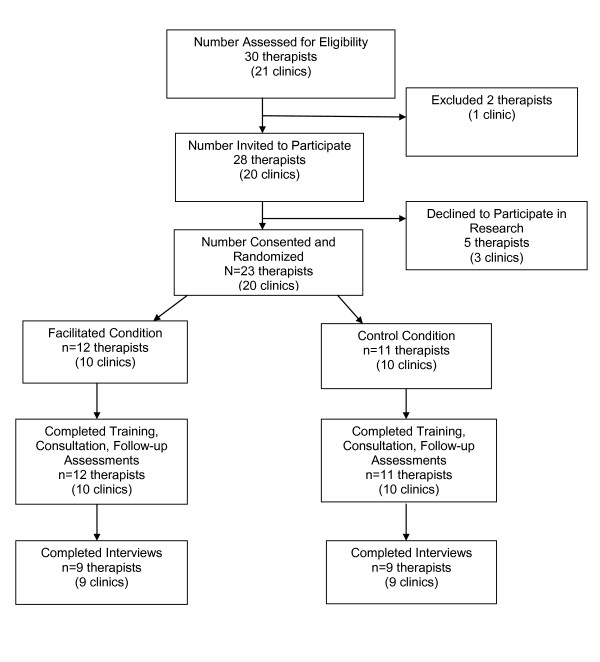
**Participant flow from eligibility to final assessment**.

All study procedures were approved by two institutional review boards, and consent to participate in the research study was obtained prior to the training. Potential clinic sites in Veterans Integrated Service Network 16 were identified by clinical leaders at the 10 VA medical centers in order to promote EBPs in newly integrated primary care (PC) clinics. Initial sites included 10 PC clinics at six VA medical centers and four outpatient community clinics and an additional 11 MH clinics in 11 VA community clinics in an effort to expand EBPs to rural clinics, where MH services are limited, and staff have few opportunities to participate in training efforts. These 21 clinics represent the total number of clinics whose participation in this study was supported by clinical leadership. From these 21 clinics, clinical leaders nominated 30 therapists to receive training and provide CBT. Nominated therapists were interviewed individually by the study Principal Investigator (PI) to explain the study and gauge the therapist's interest in delivering CBT after the training. Based on their stated interest to provide CBT, 28 therapists representing 20 clinics were invited to participate. Twenty-three (88%) therapists consented to participate. See Figure 1.

### Design

This study followed the continuous quality improvement (CQI) process [24,25], which identifies brief, clear steps for identifying the causes of the problem and potential solutions and for implementing an intervention. In our case, the problem -- to provide more CBT for depression -- was selected by network clinical leaders who knew the evidence base for CBT [26] and stated that CBT was not routinely delivered. The study team, which included frontline clinicians, speculated -- consistent with the view of clinical leaders -- that several provider-related factors might contribute to low or nonexistent CBT use in the targeted clinics, including lack of formal training in CBT or training in the distant past, limited knowledge about CBT and how to adapt it to medical settings, variable experience with CBT, comfort with current practices, resistance to change, and pressure to meet heavy workload demands. To address these issues, we chose to provide formal training and supervision in brief CBT (nine or less sessions). Full-course CBT (12 or more sessions) seemed impractical in medical settings and rural clinics because of issues of access to care and availability of MH specialty services. Additionally, it is unnecessary given that brief CBT sessions have been effective for treating depression in PC settings [27-31]. Follow-up case consultation, during which clinicians receive feedback from experts on their application of CBT, was expected to increase learning and skill. We expected variable support for practice change from clinical leaders across sites due to competing demands. To address this issue, we maintained regular communication with clinical leaders in order to quickly respond to systems barriers when identified by the facilitator.

In the current study, we employed a mixed methods, quasi-experimental approach of pairing the 20 clinics to control for at least some of the potentially many contextual differences. We matched each clinic on facility type (medical center or community clinic), clinic type (PC or MH), and relative clinic size based on staff-to-patient ratio and encounter data obtained from the network MH office. The 10 matched clinic pairs were reviewed and approved by the network MH manager, who was familiar with all sites. One clinic in each pair was randomly assigned to the facilitation condition. Randomization occurred prior to collection of baseline data and training. Twelve therapists were located at facilitated sites, and 11 therapists were located at control sites (n = 23). At follow up, qualitative interviews were conducted with nine therapists in each condition in order to better understand therapists' experience implementing CBT and identify unanticipated obstacles.

### CBT training

Training consisted of a didactic and experiential workshop (1.5 days) with biweekly phone consultations with trainers for three months after the workshop. The workshop, held in Houston TX in May 2008, was led by two CBT experts. The consultation calls were led by six experts, who were present at the workshop. A description of the training and its evaluation appears elsewhere [32]. In brief, the training focused on use of CBT modules delivered in nine or less sessions within an MH or an integrated PC setting. Workshop content included, but was not limited to, an introduction to brief CBT, use of consultation, the therapeutic relationship, case conceptualization, orienting the patient to therapy, goal setting, agenda setting, homework, identifying and responding to maladaptive thoughts, and behavioral activation. Standardized patient vignettes were employed throughout the training for practice exercises. Each trainee was provided a *Therapist's Guide to Brief CBT *[33], which contained the workshop content in a manual format. The workshop evaluation consisted of assessments of the trainers, program content, and learning environment.

As a continuation of training, trainees were asked to attend biweekly, one-hour consultation calls with a CBT expert for three months. The calls were designed to provide therapists real-time consultation on use of CBT in actual clinical encounters. Group consultation also provided the opportunity for more practiced CBT users to share their experience but focused only on the techniques and practice of 'doing' CBT. Consultants were given explicit instructions to refrain from addressing issues related to barriers and/or facilitators for implementing CBT in their setting. To ensure that therapists did not 'crosstalk' with each other, facilitated and control therapists were assigned to separate consultation groups.

### The facilitation intervention

In addition to training, 12 therapists at 10 sites received facilitation. The facilitator met with them in person or by telephone or email before and during the workshop and at least monthly (twice the first month) after the workshop for six months. The facilitator (TAT) had an education and public health background (DrPH), but by design was not an expert in CBT or a clinician. The facilitator was trained by the first author, who is an experienced facilitator in multi-site, complex behavioral adoption projects [19]. Although the facilitator was located at one site where facilitation took place, the individual was not in MH and functioned as an external facilitator for all facilitated sites.

The facilitator's tasks and interventions varied by the phase of the project and by the needs of individual therapists (Table 1). We viewed application of CBT training and development of skill competency as complex, developmental tasks that would require the facilitator to employ a range of enabling strategies varying with the therapist's self-efficacy, skill competency, and situation.

**Table 1:** Facilitator interventions by project phase

Interventions	Pre-Workshop	Workshop	**Post-workshop Months**:					
			1	2	3	4	5	6
Develop rapport with therapists and answer questions	X	X	X					
Provide education about facilitation and its benefits	X	X	X	X				
Identify goals for participating in this training	X	X	X	X				
Anticipate obstacles in meeting goals		X	X	X	X	X	X	X
Provide general encouragement and praise		X	X	X	X	X	X	X
Review goals and assess progress			X	X	X	X	X	X
Provide feedback on goal attainment			X	X	X	X	X	X
Use email reminders of calls and study deadlines				X	X	X	X	X
Provide opportunities for social comparison and support		X	X	X	X	X	X	X
Employ motivational interviewing techniques to encourage rapid application of CBT		X	X	X	X	X	X	X

Prior to the workshop, the facilitator held two conference calls with the 12 therapists to introduce the concept of facilitation and begin to develop rapport. At the workshop, the facilitator met with the 12 therapists and addressed topics related to the facilitator's role (*e.g.*, will the facilitator evaluate my job performance?), benefits of facilitation, project expectations for therapists (*e.g.*, attend facilitation calls, conduct CBT after the workshop), and anticipated barriers to conducting CBT and potential solutions. Initial post-workshop facilitation calls focused on setting individual goals for CBT implementation, attempting CBT quickly, and reinforcing all efforts to get started. The facilitator solicited barriers to getting started and helped to generate possible solutions. Later calls focused on maintaining motivation and overcoming barriers to achieving individual goals, such as challenges to providing weekly therapy sessions. In addition to scheduled calls, the facilitator received and responded to individual queries via email or telephone and sent email announcements and reminders to the group. The facilitator maintained a detailed time-log of all facilitation activities, including contacting the therapists and responding to queries.

### Study measures

#### Primary outcome

All therapists completed study surveys before the workshop (after site randomization) and at six-month follow up. Change in CBT use, our primary outcome, was assessed by self-report of percent clinical time spent conducting CBT in the past 30 days at baseline and at follow up. Estimated time spent conducting other psychotherapies was also assessed but is not reported here. Study therapists were full-time clinicians who reported spending most of their clinical time providing treatment. Although we attempted to assess implementation of CBT by tracking coded psychotherapy notes through administrative data, we were unable to implement this measure and had to abandon it (see Discussion).

#### Secondary outcomes

To further understand facilitation, the facilitator logged all contact with therapists and time spent in facilitation activities. At follow up, therapists rated the characteristics of the facilitator and the usefulness of facilitation. Therapist engagement in facilitation was assessed by the number of contacts and the time spent with the facilitator. Total time in minutes spent by each therapist in various activities with the facilitator was calculated from the log. Activities included both group and individual contacts with the facilitator, including one face-to-face meeting at the training, eight conference calls (two prior to training), individual phone calls, and email exchanges. Total number of facilitator contacts (events) with the therapists, including all calls and email exchanges, was also determined from the log.

Estimated costs of facilitation were based on the facilitator's time-log of direct contacts with therapists in minutes multiplied by salary. Therapists' salaries were calculated by estimating the average salary for clinical social workers and psychologists in the VA. Annual salaries for therapists involved in CBT facilitation were expressed as hourly rates (annual salary/52 weeks/40 hours). The estimated average hourly rate for social workers was $26.68, based on all 10 steps for General Schedule (GS) federal pay scale 10 and GS-11. For psychologists, the estimated average hourly rate was $39.40, which was based on all 10 steps for GS-12 and GS-13. The hourly rate for the facilitator was $35.47, equivalent to a GS-12, step 5. In total cost calculation, we also added fringe benefit in the amount of 24% of base salary. This information was extracted from the locality pay tables effective 2008, prepared by the US Office of Personnel Management. We also included the facilitator's travel and lodging costs incurred as a result of his visit to the CBT training workshop. These were included as facilitation costs because the facilitator undertook this effort to meet with the facilitated therapists in person to enhance rapport and the effectiveness of facilitation.

To investigate the effect of the individual characteristics of therapists on CBT adoption and use, we obtained information about therapists' formal training in CBT, and the influence that empirically based treatments have on their current practice (1 = none at all to 7 = very much). Therapists completed post-training self-efficacy ratings of their perceived understanding of the theory and concepts of CBT, acquisition of CBT skills, and willingness to conduct CBT as trained (1 = not at all to 7 = extremely). They also rated the influence of barriers (*e.g.*, lack of time/heavy caseload) on conducting CBT at their site at the end of the study (1 = not at all to 7 = very much).

### Statistical analyses

When comparing groups (*e.g.*, conditions, site types, *et al.*) on nominal-level data, chi-square tests were used. For mean comparisons, we primarily used *t *or *F *tests in an analysis of variance (ANOVA) context. We used rank correlations to evaluate the relationship among pairs of variables. All tests were two-sided, and statistical significance refers to *p *< 0.05. No power analysis was calculated for this pilot study because our sample was limited to 20 clinics.

### Qualitative methods and analysis

Qualitative methods were employed to better understand therapists' attempts to adopt CBT and identify unanticipated barriers to CBT use. At the end of the study, two study personnel conducted 30-minute, semi-structured interviews with 18 therapists to explore a range of factors that might have affected CBT use. Interview questions focused on the therapist's experience conducting CBT after the training, their clinic structure and patient population, changes in duties since the training, difficulties with documentation, and patients' response to CBT. The interviews were transcribed, and categories of barriers to conducting CBT were identified.

## Results

### Participant characteristics

Of the 23 participating therapists, 18 were women, 14 were social workers, 17 were located in a community clinic, and one-half had been practicing for eight or more years. Two-thirds reported having received previous training in CBT since graduate school. (We did not solicit the type of training, which could have ranged from a formal lecture to an intensive training plus supervision). At baseline, only five therapists reported that they were not providing any CBT; the other 18 reported spending, on average, about one quarter of their clinical time conducting CBT. More therapists reported providing CBT than reported post-graduate CBT training, although these therapists could have received CBT training in graduate school. Post-graduate training was unrelated to CBT use at baseline. Therapists with post-graduate CBT training had a mean percent usage of 26.2 compared with 23.9 for those without post-graduate CBT training (*t*[21] = 0.23, *p *= 0.82). Other therapist characteristics are provided in Table 2.

**Table 2:** Participant demographics

	Facilitated (n = 12)	Control (n = 11)	Total (n = 23)
Women	11 (92%)	7 (64%)	18 (78%)
Discipline			
Psychologists (PhD/PsyD)	3 (25%)	5 (45%)	8 (35%)
Social workers (MSW/LCSW)	9 (75%)	5 (45%)	14 (61%)
Nurses (RN)	0 (0%)	1 (9%)	1 (4%)
Medical center clinic	3 (25%)	3 (27%)	6 (26%)
Years as a therapist Mean (SD)	9.1 (7.5)	9.7 (6.0)	9.4 (6.7)
Post graduate training in CBT	7 (58%)	7 (70%)	14 (64%)
Ever used manualized therapy	3 (25%)	6 (55%)	9 (39%)
Providing CBT at baseline	8 (67%)	10 (91%)	18 (78%)
Est. % time providing CBT in the past month Mean (SD)	19.3 (22.2)	31.9 (23.7)	25.3 (23.3)

The two groups differed at baseline in some important ways. Social workers made up a bigger proportion of the facilitated group (9/12 versus 5/11 in control; chi-sq[1] = 2.10, *p *= 0.15), and psychologists were disproportionately represented in the control condition (5/11 versus 3/12 in facilitated; chi-sq[1] = 1.06, *p *= 0.30). Also, control therapists reported spending more time providing CBT (average 45 hours per month or nearly one-third of monthly clinical hours) at baseline than facilitated therapists (average 39 hours or about one-quarter of monthly clinical hours; *t*[18] = 0.41, *p *= 0.69).

### Training evaluation

Therapists' average ratings of the CBT workshop, training content, and trainers ranged from 4.2 to 4.7 (on a 5-point scale, where 4 = very good and 5 = excellent). Therapists rated the practicality of the workshop at 3.9 (3 = good, 4 = very good). Therapists attended an average of 3.2 post-training case consultation calls (out of six calls). Ratings of the consultation experience were generally high (4.1, on a 5-point scale, where 4 = very good). Ratings of therapists' understanding of CBT, their skills, and their ability to conduct CBT as trained were uniformly high and did not differ between conditions. See Table 3.

**Table 3:** Therapist characteristics and barriers to implementation of CBT by condition

	Facilitated condition Median (Q1, Q3)	Control condition Median (Q1, Q3)	Fisher's Exact Test
Influence of empirical treatments on actual practice (1 = none, 5 = very much)	3.5 (3.0, 4.0)	4.0 (3.0, 5.0)	*p *= 0.31
Self efficacy ratings			
Understanding of the theory and concepts behind CBT (1 = not at all, 7 = extremely well)	6.0 (4.5, 6.5)	7.0 (6.0, 7.0)	*p *= 0.10
Perceived skills to conduct CBT (1 = none, 7 = extremely good)	6.0 (4.5, 7.0)	7.0 (6.0, 7.0)	*p *= 0.99
Perceived ability to conduct CBT as trained (1 = not at all, 7 = extremely good)	5.5 (4.0, 6.5)	6.0 (5.0, 7.0)	*p *= 0.40
Barriers to implementation			
Lack of time/heavy caseload	5.5 (3.5, 6.0)	5.0 (2.0, 7.0)	*p *= 0.40
Patients not interested in CBT	4.0 (1.5, 5.0)	2.5 (1.0, 5.0)	*p *= 0.67
Lack of administrative support	1.0 (1.0, 2.5)	2.0 (1.0, 4.0)	*p *= 0.10
Lack of space for CBT sessions	3.0 (1.0, 6.0)	2.0 (1.0, 3.0)	*p *= 0.67
Low personal motivation to conduct CBT	2.0 (1.0, 3.0)	1.0 (1.0, 2.0)	*p *= 0.41

### Effect of facilitation

#### Change in CBT use

**Figure 2:**
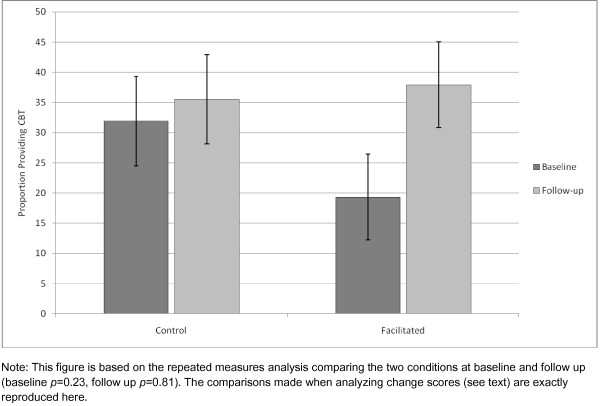
**Self-reported use of CBT from baseline to follow up across conditions**.

**Figure 3:**
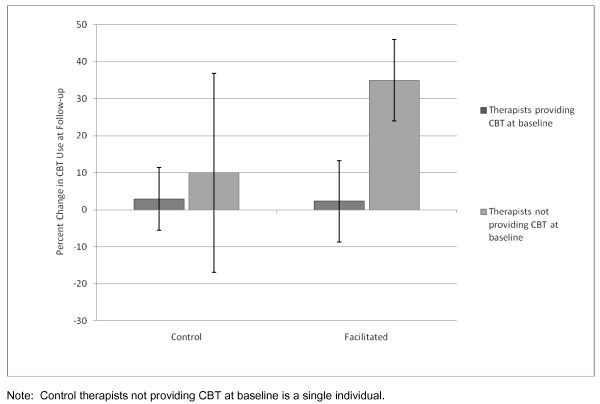
**Percent change in reported use of CBT at follow up by baseline use of CBT across conditions**.

The primary outcome was the change in CBT use an individual experienced between baseline and study end. This measure was approximately normally distributed and accounted for the correlation for the pair of observations (baseline and study end) coming from the same person. We initially compared conditions (facilitated versus control) in an ANOVA, treating matched pairs as a random effect. When the variance among pairs was estimated to be zero, it was dropped from the ANOVA. The conditions did not statistically differ when comparing mean change (pre- to post-study) in self-reported CBT use (*t*[21] = 1.27, *p *= 0.22). Employing a repeated measures analysis of the CBT percent usage data, we compared the two conditions at baseline and follow up and also found no statistical difference at either time point (*t*[21] = 1.23, *p *= 0.23 and *t*[21] = 0.24, *p *= 0.81, respectively) (Figure 2). However, the trend for the facilitation group was clearly in the hypothesized direction, with CBT usage increasing by 18.7 percentage points [95% CI: (1.3, 35.7); *t*[21] = 2.28, *p *= 0.03] from baseline for facilitated therapists; whereas the control therapists experienced only a slight increase of 3.5% [95% CI: (-14.3, 21.4); *t*[21] = 0.41, *p *= 0.68]. Therapists who were not conducting CBT at baseline showed the greatest change in CBT usage, with facilitated therapists demonstrating a 35% increase compared to a 10% increase by the single control therapist who was not providing CBT at baseline (Figure 3). Therapists who were providing CBT at baseline showed the least change (2.3% for facilitated group, 2.9% for controls). These increases translate to about 27.7 additional hours of CBT per month among facilitated therapists at follow up but only about 5.2 additional hours of CBT per month for control therapists. Estimated hours of CBT were calculated with the following formula, assuming 34 clinical hours per week: proportion of clinical time spent in CBT X 34 clinical hours/week X 4.35 weeks/month.

### Facilitation process measures

Facilitated therapists viewed the facilitator as empathetic, supportive, and responsive (average ratings were 6.4 on a 7-point scale). Therapists also indicated that the facilitator was helpful in their efforts to employ CBT (mean = 4.9, SD = 2.0). Total amount of time spent in facilitation by therapists was 26 hours and 34 minutes, with a mean of about two and one-quarter hours (133 minutes) per therapist. Total number of contacts (events) with the facilitator were 189, including conference calls, phone calls, and email exchanges, with a mean of 16 contacts per therapist. On average, three therapists attended post-workshop facilitation calls, although these calls represented only a portion of contacts with the facilitator. Of the 12 therapists who reported CBT use at baseline, six were least likely to participate on facilitation calls. Approximately one-half of the total facilitation time (735 minutes, 46.1%) was used by three therapists.

When we calculated correlation coefficients with different sets of variables, we found no significant associations between change in CBT usage and total time in facilitation or number of facilitator contacts with therapists. There was a negative, suggestive relationship between total time in facilitation and any postgraduate training in CBT (*r *= -0.67, *p *= 0.07). Specifically, therapists who had postgraduate training in CBT spent less time in facilitation activities.

### Cost estimates

The facilitator's direct contacts with therapists totaled 10 hours, 28 minutes. The facilitator also spent 14 hours and 38 minutes in support activities, such as reading and writing emails, making phone calls, and researching questions. Altogether, the facilitator spent 25 hours, 6 minutes in facilitation-related activities. Total salary, fringe benefits, and travel costs for the facilitator were calculated to be $1,445.47. In addition, facilitated therapists spent a total of 26 hours, 34 minutes in direct contact with the facilitator. Given therapists' discipline, their approximate total salary and fringe benefits, costs for the time spent in facilitation were $1,013.63. The total costs for the facilitator's and 12 therapists' time spent in activities associated with facilitation were $2,458.80 over seven months, for a benefit of about 28 additional hours of CBT per month per therapist or about 332 more hours of CBT a month for the 12 therapists receiving facilitation.

### Provider characteristics and increased CBT use

Provider location (medical center versus community clinic) and discipline were both unrelated to increased CBT usage (*t*[21] = 0.01, *p *= 0.94 and *t*[21] = 0.01, *p *= 0.93, respectively). Post-graduate training in CBT was also unrelated to increased CBT usage (*r *= -0.03). Fourteen therapists reported at least some postgraduate training in CBT; eight of these increased CBT use by follow up. Of the nine therapists having no post-graduate CBT training, six increased their CBT use over time. The largest gains in CBT use were among facilitated therapists who had no post-graduate training in CBT (20% increase) compared with control therapists with no previous training in CBT (7.5% increase). Increased use of CBT was not correlated with the perceived influence of evidence based treatments on actual practice or to understanding CBT, learning CBT skills, or conducting CBT as trained. No barriers were significantly associated with increased CBT use.

### Qualitative results

The post-study interviews revealed several unanticipated barriers to using CBT. Four common themes emerged, as follows: lack of control over the clinic schedule; rejection of CBT as a treatment option; therapist duties; and poor communication between therapists and clinical leadership. At follow up, some therapists reported that they could not schedule patients for recurrent sessions because they lacked control over their schedule, although in only two cases had this been raised as a problem during the study. Clinics could be completely scheduled several weeks in advance; or, in the case of open-access clinics, scheduling regularly occurring therapy sessions was not possible. Inability to block one's schedule was also identified as a barrier to participation on facilitation calls, although clinical leaders had agreed to support therapists' participation. Some therapists also rejected CBT as a treatment option because they found it to be too structured or difficult to implement as trained, or some decided that they did not want to change their current practices. Others stated that older veterans and veterans with chronic post-traumatic stress disorder, in particular, were uninterested in CBT. Some therapists found it difficult to get patients to commit to regular sessions because of the distance to clinic or an unwillingness to miss work. In addition, a few therapists noted that their current duties (*e.g.*, only intake assessments) or changes in duties prevented or reduced the likelihood of conducting psychotherapy. However, the most common theme among therapists was poor communication with their clinical leaders. Despite strong requests to clinical leaders and participating therapists to meet before training and again after training to discuss local expectations for providing CBT, none had done so.

## Discussion

To our knowledge, this is the first controlled study of external facilitation as an implementation strategy for an EBP. Consistent with our hypothesis, facilitated therapists demonstrated markedly increased use of CBT from baseline compared to controls (19% versus 4%). The increase in CBT usage by facilitated therapists is unlikely to be caused by training alone. Facilitated therapists who were not conducting CBT at baseline evidenced a larger gain in CBT use (35%) than the single control therapist who was not conducting CBT at baseline (10%). Therapists who were providing CBT at baseline, no matter which condition, showed the least change. These findings lend support to the notion that facilitation may enhance the adoption of a complex practice such as psychotherapy.

Facilitation was likely to have influenced therapists in different ways. Some therapists were already providing some CBT, and these individuals attended few or none of the post-workshop facilitation calls yet still reported increased CBT use. For them, the training and facilitator's presence (*e.g.*, pre-workshop meeting, regular email contacts) may have served as a booster to expand use of CBT. A few therapists made regular use of the facilitation calls, and others attended occasionally. For them, facilitation may have provided support for CBT adoption beyond that provided by the consultation calls. For about 28 additional hours of CBT per month per therapist at follow up for the 12 facilitated therapists, the facilitator spent about 25 hours over seven months at a total cost of less than $2,500 (about $351 per month). If facilitated therapists provided CBT at about the same rate for just the last half of the study, the cost for each additional hour of CBT would be about $2.47. The 11 control therapists gained only about five hours of CBT per month per therapist by follow up for the cost of training alone (which we did not calculate). These results suggest a moderate return on investment for facilitation. Training costs were not included in our analyses because training was consistent across groups, and we were interested in the effect of facilitation above training alone.

We matched sites on some organizational variables because we expected that contextual variables would influence implementation more than characteristics of the therapist. However, in our quantitative analyses the variability in CBT use attributable to organizational characteristics appeared to be negligible. It is important to note, however, that the contextual measures we used were crude (*e.g.*, relative size of clinic) rather than a direct measure of specific organizational characteristics that are known to influence implementation, such as quality and strength of leadership and organizational culture. Because we used only three contextual characteristics, there were many organizational factors left unmeasured. For example, our qualitative results indicated that some aspects of the clinic's policies and practices presented barriers to implementation. Three barriers in particular (lack of control over the clinic schedule, conflicts with other duties, poor communication with leadership) appear to be important organizational issues that we did not measure. In future studies of implementation at the clinic level, these factors may be especially important to assess. Meanwhile, attention to these issues during implementation will be critical to the success of any implementation effort.

This pilot study has several limitations. The primary measure of implementation was based on self-report. We attempted to measure implementation by tracking progress notes coded in charts for brief CBT, but our attempt failed. We gave therapists two procedures for coding progress notes for brief CBT so that we could track them. A specially designed CBT note template in the medical records automatically coded the note. The template was brief and included checklists to denote the problem and patient presentation and open-text fields for session content and the treatment plan. If therapists chose not to use the template, the progress note could be coded manually in the encounter section. After the training, therapists were reminded how to code notes, and they acknowledged their understanding. Yet, therapists rarely used the template or coded notes but still reported to the facilitator and the consultants that they were conducting CBT. The qualitative interviews revealed that therapists found locating and completing the template or manually coding the note to be a significant departure from routine practice and highly burdensome. Had we spent more time obtaining support for new documentation procedures, perhaps our efforts would have been more productive. However, we were forced, ultimately, to rely on self-report alone. We did not assess what therapists meant by saying they provided CBT. Anecdotal comments suggest that some therapists who reported conducting CBT were using CBT techniques, not full CBT. Some therapists showed a marked decline in CBT use at follow up, which may have been related in part to their redefinition of what constitutes CBT.

The use of individual techniques or specific skills, rather than a comprehensive therapeutic intervention, is a critical issue for implementation of EBPs because we do not know to what extent such treatment is true to the model of CBT that has been shown to be effective. Others have found similar patterns in terms of how therapists employ evidence-based training in routine practice. A recent web-based survey of 2,607 US and Canadian psychotherapists found that most stick to the treatment approach that they were trained in and prefer to adopt selected techniques from other psychotherapies [34]. Many therapists describe their approach to psychotherapy as 'eclectic' [35].

Our ability to find a difference between conditions was reduced by our small sample size and because several therapists in both conditions were spending about 25% of their time providing CBT at baseline, despite our attempt to enroll therapists who were conducting little or no CBT. The selection of clinics and therapists was also based on clinical leader's nomination, which may have introduced a bias. Leaders' consent is necessary for clinic participation. However, after pairs of clinics were matched, facilitation was randomized within the pairs, effectively eliminating any bias on facilitation effects. Further, leaders appear to have been not well engaged, despite regular contact with them. No reporting bias was evident among therapists. Some therapists actually decreased their reported use of CBT over time, and in general therapists in the control conditions showed only a negligible increase in CBT. Finally, many important factors related to implementation, especially at the organizational level, were not assessed in this pilot. Future studies of the effect of facilitation on use of EBPs should include larger samples and measure a fuller range of both individual and organizational factors.

## Summary

In conclusion, our pilot study suggests that external facilitation is a promising, low-cost strategy to promote implementation of a complex, evidence-based psychotherapy, CBT, in routine clinical care. This strategy appears to have been effective, even though we did not address some key organizational barriers to implementation. It is possible, however, that the effects of facilitation may decay over time, given the myriad clinical demands clinicians face in routine practice settings. Some type of 'booster' facilitation sessions may be needed to sustain the early benefits of external facilitation.

## Competing interests

The authors declare that they have no competing interests in the conduct of this study.

## Authors' contributions

MRK, GS, DB, JAC, QS, and TAT contributed to study design. MRK drafted and revised the manuscript. JAC led the training. TT served as the facilitator. DB and RDL conducted the data analyses. All authors read, contributed to, and approved the final manuscript.
